# Roles of noncoding RNAs in chronic obstructive pulmonary disease

**DOI:** 10.2478/jtim-2023-0084

**Published:** 2023-07-05

**Authors:** Xin Qiao, Yuxiao Ding, Abdullah Altawil, Yan Yin, Qiuyue Wang, Wei Wang, Jian Kang

**Affiliations:** Department of Pulmonary and Critical Care Medicine, the First Hospital of China Medical University, Shenyang 110001, Liaoning Province, China

## Introduction

Chronic obstructive pulmonary disease (COPD) is a chronic heterogeneous disease characterized by persistent airflow obstruction and variable clinical presentations.^[1,2]^ A lack of understanding regarding the molecular mechanisms underlying COPD makes the identification of critical molecules involved in COPD crucial for the development of novel diagnostic measures and therapeutic strategies. In recent decades, wide-ranging profiling methods such as microarrays and next-generation sequencing have made it easier to identify RNA transcripts that do not encode proteins, referred to as noncoding RNAs (ncRNAs).^[3]^ NcRNAs comprise a diverse range of RNA species, characterized according to their length, shape, and location. Many ncRNAs are involved in epigenetic and posttranscriptional gene regulation, including microRNAs (miRNAs), tRNA-derived small RNAs (tsRNAs) and PIWI-interacting RNAs (piRNAs).^[4]^ Long noncoding RNAs (lncRNAs) and circular RNAs (circRNAs) can fold into complex secondary structures that facilitate their interactions with DNA, RNA, and protein.^[4]^ Additionally, lncRNAs and circRNAs can bind to miRNAs in a competitive endogenous RNA (ceRNA) network that prevents targeted mRNA degradation.^[5,6]^ Recent studies have shown that ncRNAs play crucial roles in multiple pathophysiological processes associated with COPD.^[5,7,8]^ A better understanding of the role of ncRNAs in COPD could contribute to the detection of biomarkers and the identification of new therapeutic targets. Here, we summarize the current findings regarding the potential role of ncRNAs, especially miRNAs, lncRNAs, and circRNAs. Additionally, we propose considerations regarding present and future research in this area.

## Ncrna Dysregulation Contributes to Copd Progression

**Figure 1:**
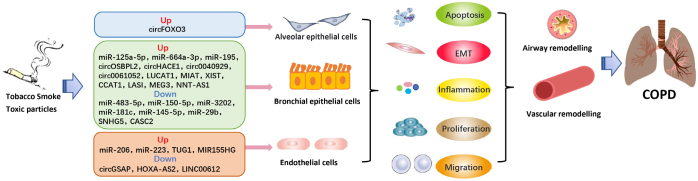
NcRNAs play a crucial role as regulators in the pathophysiological processes of COPD. COPD: chronic obstructive pulmonary diseae; EMT: epithelial mesenchymal transformation.

**Figure 2:**
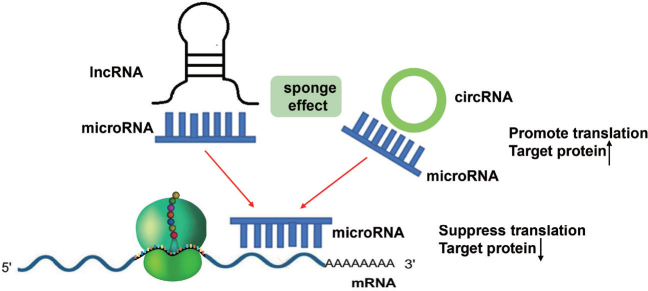
LncRNAs and circRNAs can act as sponges for microRNAS. By binding to these microRNAs, they prevent microRNAs from binding to their target mRNAs, thereby abolishing post-transcriptional regulation.

Emerging evidence suggests that differentially expressed ncRNAs participate in the regulation of proliferation, apoptosis, invasion, epithelial–mesenchymal transition (EMT), and inflammation in multiple relevant cell types, contributing to the pathophysiological changes in COPD (Figure 1). Supplementary Table 1 summarizes the list of miRNAs, circRNAs, and lncRNAs with their targets and functions in COPD. Notably, in the lncRNA/ circRNA-miRNA-mRNA networks, lncRNAs and circRNAs could sponge miRNAs as ceRNAs, inhibit miRNA expression, and enhance the translation of target mRNA (Figure 2). For instance, lncRNA cancer susceptibility candidate 2 (*CASC2*)^[9]^and circRNA HECT domain and ankyrin repeat containing E3 ubiquitin protein ligase 1 (*circHACE1*) ^[10]^ bind to *miR-18a-5p* and *miR-485-3p*, respectively, to participate in human bronchial epithelial (16HBE) cell apoptosis and inflammation in response to cigarette smoking extract (CSE). Furthermore, Sundar *et al*.^[11]^ discovered a small number of differentially expressed piRNAs and tsRNAs among nonsmokers, smokers, and COPD patients from RNA sequencing data. However, the functional role of piRNAs and tsRNAs in COPD remains unclear.

In summary, given the wide range of regulatory mechanisms and the diversity of downstream pathways affected, many ncRNAs and their crosstalk have been found through *in vivo* and *in vitro* experiments to be important contributors to COPD progression and regarded as possible clinical relevance.

## Ncrnas Can Be Used as Adjunct Biomarkersfor Copd Diagnosis and Prognosis

It has been found that differentially expressed ncRNAs can be detected in human samples such as sputum, plasma, or serum, and these may serve as adjunct biomarkers for COPD diagnosis and prognosis. For example, circ0040929,^[12]^ circRNA oxysterol binding protein like 2 (*circOSBPL2*),^[13]^ lncRNA lung cancer associated transcript 1 (*LUCAT1*),^[14]^ and *miR-125a-5p*^[15]^ show a more sensitive expression pattern in smokers with COPD than in those without COPD. *LUCAT1* ^[16]^ expression levels are correlated with inflammation in COPD patients, and *circRNA0001859*,^[17]^ *hsa-miR-664a-3p*,^[18]^ lncRNA small nucleolar RNA host gene 5 (*SNHG5*),^[19]^ and *CASC2*^[9]^ are correlated with airflow limitation severity. Furthermore, lncRNA antisense non-coding RNA at the INK4 locus (*ANRIL*)^[20]^ expression is decreased in COPD patients, especially those with acute exacerbations (AECOPD), while lncRNA nuclear-enriched abundant transcript 1 (*NEAT1*)^[21]^ expression is increased. The area under the curve (AUC) indicates that lncRNAs *ANRIL* and *NEAT1* can distinguish stable COPD patients from AECOPD patients, as well as predict COPD susceptibility and acute exacerbation risk.^[20,21]^ Moreover, the expression levels of the lncRNAs *ANRIL* and *NEAT1* are both correlated with inflammatory cytokines (tumor necrosis factor α [TNF-α], interlenkin [IL]-1β, and IL-17A) and Global Initiative for Chronic Obstructive Lung Disease (GOLD) stage in COPD patients,^[20,21]^ suggesting that they are potential biomarkers of COPD progression.

In addition, noncoding RNA (ncRNA) is useful in diagnosing comorbidities of COPD. For instance, the lower level of *circRNA0001859* in serum can be used to identify patients with lung cancer from COPD patients.^[17]^ Low plasma circRNA-gamma-secretase-activating protein (*circGSAP*) levels might be a promising diagnostic and prognostic indicator for COPD-pulmonary arterial hypertension (PAH).^[22]^ Serum *miR-1233* and *miR-134* both have higher diagnostic accuracy for AECOPD with acute pulmonary embolism (APE) than D dimers.^[23]^ In addition, the role of certain ncRNAs in indicating COPD comorbidities is unspecified and paradoxical. A recent review discusses that lncRNA maternally expressed gene 3 (*MEG3*), lncRNA OPA-interacting protein 5 antisense transcript 1 (*OIP5-AS1*), and ln-cRNA taurine upregulated gene 1 (*TUG1*) are involved in asthma and COPD pathogenesis by sponging different miRNAs.^[5]^ Further research into the ncRNAs mentioned above as biomarkers for the diagnosis of COPD overlapping with asthma is warranted. Wang *et al*.^[24]^ previously demonstrated that *TUG1* was significantly upregulated in patients with PAH and that *TUG1* knockdown significantly prevented the development of PAH *in vivo*, suggesting that *TUG1* may also be a novel and promising biomarker for COPD complicated with PAH. Moreover, *MiR-223*^[16]^ and *NEAT1*^[25]^ both have promoting roles for lung cancer carcinogenesis and COPD, and whether they are also involved in COPD complicated with lung cancer remains to be determined. It is worth mentioning that the blood levels of *MEG3* are higher in COPD patients but lower in non-small-cell lung cancer patients.^[26,27]^ Thus, the role of *MEG3* in COPD with lung cancer requires further research and validation.

As noted above, in COPD, most biomarker studies related to ncRNAs (especially lncRNAs, miRNAs, and circRNAs) have focused on distinguishing COPD patients from non-COPD patients or predicting disease severity and comorbidities. It is well known that COPD is a chronic airway disease with high heterogeneity. The role of ncRNAs in determining the phenotype and endotype of COPD and in predicting the response to specific treatments (*e.g*., glucocorticoids) needs further investigation. In addition, no single ncRNA or ncRNA panel has passed the test as an analytically validated and clinically useful biomarker. Attempts have been made through meta-analysis to assess the diagnostic accuracy of various ncRNAs in COPD.

## The Therapeutic Potential of Ncrnas for Copd

NcRNAs have been demonstrated to play a crucial role as regulators in the pathophysiological processes of COPD. The pharmacological action of ncRNA-based therapies has been demonstrated to target proliferation, apoptosis, inflammation, and migration as potential therapeutics for COPD, including small interfering RNAs, short hairpin RNAs, miRNA mimics, and anti-microRNAs. In COPD mouse models, intranasal administration of anti-*miR-195* lentiviruses or *miR-181c* mimics attenuates neutrophil and macrophage infiltration, lung parenchymal destruction, and levels of proinflammatory factors in bronchoalveolar lavage fluid (BALF),^[28]^ while intranasal delivery of short hairpin RNA (shRNA) lentivirus against *TUG1* blocks cigarette smoking (CS)-induced inflammation and remodeling.^[29]^ CS-induced increases in neutrophils, macrophages, and BALF cells could also be significantly reduced with lentivirus-based knockdown of circRNA forkhead box O3 (circFOXO3) *in vivo*.^[30]^ *in vitro* studies have shown that *miR-206* antagomirs or *miR-483-5p* mimics enhance vascular remodeling and fibrosis,^[31,32]^ whereas *miR-27-3p* antagomirs or *miR-3202* mimics inhibit inflammation in response to CSE.^[33,34]^ Using small interfering RNA (siRNA) to knockdown MIR155 host gene (*MIR155HG*) results in a switch from the M1 to M2 macrophage phenotype along with reduced proin-flammatory cytokines in CSE-treated human pulmonary microvascular endothelial cells (HPMECs).^[35]^ Currently available small RNA high-throughput sequencing technology can detect tsRNAs and piRNAs; however, few have been functionally characterized in COPD models for further validation of potential therapeutic targets.

To the best of our knowledge, ncRNAs as therapeutic targets (*e.g*., MRX34, a *miR-34a* mimic in advanced solid tumors) for cancer have been investigated and tested in clinical trials.^[36]^ However, advances concerning the roles of ncRNAs in COPD have only been studied in cell lines or animal models, and further effort is needed to explore the feasibility of their clinical application.^[37]^

## Conclusion and Perspectives

NcRNAs, including miRNAs, lncRNAs, and circRNAs, can be detected in the serum, plasma, sputum, or urine of COPD patients and may serve as diagnostic biomarkers or prognostic indicators. Notably, the abnormal expression of these ncRNAs has been linked to the various pathophysiological processes of COPD, underscoring their feasibility as a novel therapeutic modality for COPD. In addition, there are many other less studied ncRNA classes, such as piRNAs and tsRNAs, that may warrant further exploration of their precise function and mechanism in COPD. In the past several years, studies on the crosstalk of lncRNA/circRNA-miRNA-mRNA in the pathogenesis of many diseases, including COPD, have received increasing attention; this will likely open a new horizon for the identification of therapeutic targets for COPD.

There are several challenges to overcome regarding the clinical application of ncRNAs. First, a large number of investigations have discovered various ncRNAs for identifying COPD, acute exacerbations, and comorbidities. The screening and development of the optimal ncRNA will require further studies involving larger sample sizes. Second, the development of diagnostic biomarkers found in urine, sputum, or blood would avoid the need for invasive procedures associated with tissue collection. Given that ncRNAs are often expressed at lower levels than protein-coding genes, more research is needed to identify candidates that are highly stable and easily detected in body fluids. Third, regulation of the pharmacological action of ncRNA in the COPD model is mainly dependent on small interfering RNAs, short hairpin RNAs, miRNA mimics, and anti-microRNAs. Antisense oligonucleotides (ASOs) or custered regularly interspaced short palindromic repeats (CRISPR) may also be worth exploring for the treatment of COPD. However, safety issues associated with random mutations of target sites should be assessed. If there are abnormal changes such as large fragment loss and chromosome rearrangement, the safety issues will be too risky. In addition, ncRNA drugs are usually given intravenously for cancer patients, while in COPD, direct delivery to the lungs by inhalation is the most effective way to reduce systemic adverse effects. However, the stability and economic benefits of ncRNAs must be considered and optimized. Improvements in oligonucleotide chemistry, editing efficiency and accuracy of target sites, and delivery methods should continuously be pursued in future studies to mitigate these issues. Finally, ncRNAs can regulate multiple genes simultaneously; therefore, special precautions to minimize off-target adverse effects must be made.

To conclude, there is growing evidence that ncRNAs may be useful for diagnosing and treating COPD, and a large ncRNA network is being established to explore their possible mechanisms of action in the disease. Although our understanding of ncRNAs in COPD is still at an early stage, the discovery of ncRNAs has opened a new chapter in the history of medicine, one that promises to improve the way that COPD is diagnosed and treated. It is expected that genetic diagnosis and therapeutics based on ncRNA will be widely available in the future.

## Supplementary Material

Supplementary material

## References

[1] Vogelmeier CF, Criner GJ, Martinez FJ, Anzueto A, Barnes PJ, Bourbeau J (2017). Global Strategy for the Diagnosis, Management, and Prevention of Chronic Obstructive Lung Disease 2017 Report. GOLD Executive Summary. Am J Respir Crit Care Med.

[2] Sun L, Chen Y (2022). Interpretation of the key issues of expert consensus on immunomodulatory therapies for chronic obstructive pulmonary disease. J Transl Intern Med.

[3] Xue C, Gu X, Bao Z, Su Y, Lu J, Li L (2022). The Mechanism Underlying the ncRNA Dysregulation Pattern in Hepatocellular Carcinoma and Its Tumor Microenvironment. Front Immunol.

[4] Yan H, Bu P (2021). Non-coding RNA in cancer. Essays Biochem.

[5] Qiao X, Hou G, He YL, Song DF, An Y, Altawil A (2022). The Novel Regulatory Role of the lncRNA-miRNA-mRNA Axis in Chronic Inflammatory Airway Diseases. Front Mol Biosci.

[6] Li Y, Lu X, Li W, Shi Z, Du W, Xu H (2022). The circRERE/miR-144-3p/TLR2/MMP9 signaling axis in COPD pulmonary monocytes promotes the EMT of pulmonary epithelial cells. Biochem Biophys Res Commun.

[7] Soni DK, Biswas R (2021). Role of Non-Coding RNAs in Post-Transcriptional Regulation of Lung Diseases. Front Genet.

[8] Liu P, Wang Y, Zhang N, Zhao X, Li R, Wang Y (2022). Comprehensive identification of RNA transcripts and construction of RNA network in chronic obstructive pulmonary disease. Respir Res.

[9] Liu P, Zhang H, Zeng H, Meng Y, Gao H, Zhang M (2021). LncRNA CASC2 is involved in the development of chronic obstructive pulmonary disease via targeting miR-18a-5p/IGF1 axis. Ther Adv Respir Dis.

[10] Zhou F, Cao C, Chai H, Hong J, Zhu M (2021). Circ-HACE1 Aggravates Cigarette Smoke Extract-Induced Injury in Human Bronchial Epithelial Cells via Regulating Toll-Like Receptor 4 by Sponging miR-485-3p. Int J Chron Obstruct Pulmon Dis.

[11] Sundar IK, Li D, Rahman I (2019). Small RNA-sequence analysis of plasma-derived extracellular vesicle miRNAs in smokers and patients with chronic obstructive pulmonary disease as circulating biomarkers. J Extracell Vesicles.

[12] Miao Y, Wu J, Wu R, Wang E, Wang J (2022). Circ_0040929 Serves as Promising Biomarker and Potential Target for Chronic Obstructive Pulmonary Disease. Int J Chron Obstruct Pulmon Dis.

[13] Zheng C, Zhang Y, Zhao Y, Duan Y, Mu Q, Wang X (2021). Circ-OSBPL2 Contributes to Smoke-Related Chronic Obstructive Pulmonary Disease by Targeting miR-193a-5p/BRD4 Axis. Int J Chron Obstruct Pulmon Dis.

[14] Zhao S, Lin C, Yang T, Qian X, Lu J, Cheng J (2021). Expression of long non-coding RNA LUCAT1 in patients with chronic obstructive pulmonary disease and its potential functions in regulating cigarette smoke extract-induced 16HBE cell proliferation and apoptosis. J Clin Lab Anal.

[15] Wu H, Ma H, Wang L, Zhang H, Lu L, Xiao T (2022). Regulation of lung epithelial cell senescence in smoking-induced COPD/emphysema by microR-125a-5p via Sp1 mediation of SIRT1/HIF-1a. Int J Biol Sci.

[16] Li S, Feng Y, Huang Y, Liu Y, Wang Y, Liang Y (2020). MiR-223-3p regulates cell viability, migration, invasion, and apoptosis of non-small cell lung cancer cells by targeting RHOB. Open Life Sci.

[17] Chen S, Yao Y, Lu S, Chen J, Yang G, Tu L (2020). CircRNA0001859, a new diagnostic and prognostic biomarkers for COPD and AECOPD. BMC Pulm Med.

[18] Zhong S, Chen C, Liu N, Yang L, Hu Z, Duan P (2019). Overexpression Of hsa-miR-664a-3p Is Associated With Cigarette Smoke-Induced Chronic Obstructive Pulmonary Disease Via Targeting FHL1. Int J Chron Obstruct Pulmon Dis.

[19] Shen Q, Zheng J, Wang X, Hu W, Jiang Y, Jiang Y (2020). LncRNA SNHG5 regulates cell apoptosis and inflammation by miR-132/PTEN axis in COPD. Biomed Pharmacother.

[20] Ge J, Geng S, Jiang H (2019). Long noncoding RNAs antisense noncoding RNA in the INK4 locus (ANRIL) correlates with lower acute exacerbation risk, decreased inflammatory cytokines, and mild GOLD stage in patients with chronic obstructive pulmonary disease. J Clin Lab Anal.

[21] Ming XY, Duan WZ, Yi W (2019). Long non-coding RNA NEAT1 predicts elevated chronic obstructive pulmonary disease (COPD) susceptibility and acute exacerbation risk, and correlates with higher disease severity, inflammation, and lower miR-193a in COPD patients. Int J Clin Exp Pathol.

[22] Sun Y, Wu W, Zhao Q, Jiang R, Li J, Wang L (2022). CircGSAP regulates the cell cycle of pulmonary microvascular endothelial cells via the miR-942-5p sponge in pulmonary hypertension. Front Cell Dev Biol.

[23] Peng L, Han L, Li XN, Miao YF, Xue F, Zhou C (2020). The Predictive Value of microRNA-134 and microRNA-1233 for the Early Diagnosis of Acute Exacerbation of Chronic Obstructive Pulmonary Disease with Acute Pulmonary Embolism. Int J Chron Obstruct Pulmon Dis.

[24] Wang S, Cao W, Gao S, Nie X, Zheng X, Xing Y (2019). TUG1 Regulates Pulmonary Arterial Smooth Muscle Cell Proliferation in Pulmonary Arterial Hypertension. Can J Cardiol.

[25] Ma F, Lei YY, Ding MG, Luo LH, Xie YC, Liu XL (2020). LncRNA NEAT1 Interacted With DNMT1 to Regulate Malignant Phenotype of Cancer Cell and Cytotoxic T Cell Infiltration via Epigenetic Inhibition of p53, cGAS, and STING in Lung Cancer. Front Genet.

[26] Lv D, Bi Q, Li Y, Deng J, Wu N, Hao S (2021). Long non‑coding RNA MEG3 inhibits cell migration and invasion of non‑small cell lung cancer cells by regulating the miR‑21‑5p/PTEN axis. Mol Med Rep.

[27] Zhao Y, Zhu Z, Shi S, Wang J, Li N (2019). Long non-coding RNA MEG3 regulates migration and invasion of lung cancer stem cells via miR-650/SLC34A2 axis. Biomed Pharmacother.

[28] Mei D, Tan WSD, Tay Y, Mukhopadhyay A, Wong WSF (2020). Therapeutic RNA Strategies for Chronic Obstructive Pulmonary Disease. Trends Pharmacol Sci.

[29] Gu W, Yuan Y, Wang L, Yang H, Li S, Tang Z (2019). Long non-coding RNA TUG1 promotes airway remodelling by suppressing the miR-145-5p/DUSP6 axis in cigarette smoke-induced COPD. J Cell Mol Med.

[30] Zhou L, Wu B, Yang J, Wang B, Pan J, Xu D (2021). Knockdown of circ-FOXO3 ameliorates cigarette smoke-induced lung injury in mice. Respir Res.

[31] Sun Y, An N, Li J, Xia J, Tian Y, Zhao P (2019). miRNA-206 regulates human pulmonary microvascular endothelial cell apoptosis via targeting in chronic obstructive pulmonary disease. J Cell Biochem.

[32] Shen ZY, Tang WX, Guo J, Sun SH (2017). miR-483-5p plays a protective role in chronic obstructive pulmonary disease. Int J Mol Med.

[33] Shen W, Liu J, Fan M, Wang S, Zhang Y, Wen L (2018). MiR-3202 protects smokers from chronic obstructive pulmonary disease through inhibiting FAIM2: An in vivo and *in vitro* study. Exp Cell Res.

[34] Wang D, He S, Liu B, Liu C (2018). MiR-27-3p regulates TLR2/4-dependent mouse alveolar macrophage activation by targetting PPARγ. Clin Sci(Lond).

[35] Li N, Liu Y, Cai J (2019). LncRNA MIR155HG regulates M1/M2 macrophage polarization in chronic obstructive pulmonary disease. Biomed Pharmacother.

[36] Hong DS, Kang YK, Borad M, Sachdev J, Ejadi S, Lim HY (2020). Phase 1 study of MRX34, a liposomal miR-34a mimic, in patients with advanced solid tumours. Br J Cancer.

[37] Zhang J, Han Y, Hua J, He B (2022). Inhaled antibiotics and airway bacterial decolonization for patients with chronic obstructive pulmonary disease: The rationale and future. J Transl Intern Med.

