# Supplementary material for: Roles of noncoding RNAs in chronic obstructive pulmonary disease
Source: J Transl Int Med. 2023 Jul 5;11(2):106–10. doi: 10.2478/jtim-2023-0084 (PMC10680378; doi:10.2478/jtim-2023-0084)
Supplement: Supplementary file 1 — Supplementary material [file jtim-2023-0084_sm.pdf]

Supplemental Table 1. List of noncoding RNAs and their targets and functions in chronic obstructive pulmonary disease.

Types	NcRNAs	Source	Expression	Target/pathway	Function	Study
miRNAs	miR-3202	Blood from smokers without or with COPD	down	FAIM2	Inhibited bronchial epithelial cells (HBECs) apoptosis and proinflammatory cytokine production.	(Shen *et al.*, 2018) [1]
	miR-206	Lung tissues from COPD patients	up	Notch3, VEGFA	Promoted cigarette smoking extract (CSE)-induced human pulmonary microvascular endothelial cell (HPMECs) apoptosis.	(Sun *et al.*, 2018) [2]
	miR-27-3p	Mice model	up	PPAR-γ/TLR2/4	Promoted CS/LPS-treated proinflammatory cytokine production and M1 polarization of alveolar macrophage.	(Wang *et al.*, 2018) [3]
	miR-181c	Lung samples from COPD patients	down	CCN1	MiR-181c overexpression decreased the CS-induced inflammatory response, neutrophil infiltration, ROS generation, and inflammatory cytokines.	(Du *et al.*, 2017) [4]
	miR-195	Lung tissues from COPD patients	up	PHLPP2/Akt	MiR-195 knockdown alleviated CS-induced lung injury and reduced inflammatory cell infiltration and secretion of IL-6 and TNF-α.	(Gu *et al.*, 2017) [5]
	miR-223	Lung tissues from COPD patients	up	HDAC2	Upregulated CX3CL1.	(Leuenberger *et al.*, 2016) [6]
	miR-149-3p	Blood from smokers without or with COPD	down	TLR4/NF-κB	Reducing miR-149-3p upregulated IL-1β and TNF-α.	(Shen *et al.*, 2017) [7]
	miR-145-5p	Lung tissues from smokers without or with COPD	down	KLF5	Regulated CSE-stimulated HBECs apoptosis and inflammation.	(Dang *et al.*, 2019) [8]
	miR-29b	Lung tissues and plasma from COPD patients	down	BRD4	Regulated CSE-induced IL-8.	(Tang *et al.*, 2019) [9]
	miR-483-5p	Lung tissues from COPD patients	down	α-SMA and fibronectin	Inhibited the TGF-β-mediated decrease in cell proliferation, α-SMA and fibronectin expression in BEAS-2B and HFL1 cells.	(Shen *et al.*, 2017) [10]
	hsa-miR-664a-3p	Lung tissue and PBMCs from COPD patients	up	FHL1	Positively correlates with FEV_1/FVC%.	(Zhong *et al.*, 2019) [11]
	miR-125a-5p	Lung tissues from smokers without or with COPD	up	Sp1/SIRT1/HIF-1α	Knockdown of miR-125a-5p reduced lung epithelial cell senescence and COPD/emphysema.	(Wu *et al.*, 2022) [12]
	miR-150-5p	Plasma from COPD patients	down	IRE1α	MiR-150-5p overexpression decreased CSE-treated HBE cells inflammation and ER stress.	(Zhu *et al.*, 2022) [13]
circRNAs	circRNA0001859	Serum from COPD patients	down	None	Positively correlated with FEV_1% in COPD patients.	(Chen *et al.*, 2020) [14]
	circBbs9	Mice model	up	miR-30e-5p/Adar, NLRP3	Exacerbate $PM_{2.5}$-induced inflammatory response in lung tissue.	(Li *et al.*, 2020) [15]
	circFOXO3	Mice model	up	miR-214-3p/IKK-β	Enhances CSE-induced expression of inflammatory cytokines in MLE12 cells.	(Zhou *et al.*, 2021) [16]
	circOSBPL2	Lung tissues of smokers without or with COPD	up	miR-193a-5p/ BRD4	Contributed to the apoptosis, inflammation, and oxidative stress of CSE-induced HBECs.	(Zheng *et al.*, 2021) [17]
	circ0040929	Serum from smokers without or with COPD	up	miR-515-5p/ IGFBP3	Aggregate CSE-triggered apoptosis, inflammation and airway remodeling in 16HBE cells.	(Miao *et al.*, 2022) [19]

Continued

Supplemental Table 1 Continued

Types	NcRNAs	Source	Expression	Target/pathway	Function	Study
circRNAs	circ0061052	Mice model	up	miR-515-5p/ FoxC1/Snail	Promote CS-induced epithelial-mesenchymal transition (EMT) and airway remodeling.	(Ma *et al.*, 2020) [20]
	circNFXL1_009	Whole-blood samples from COPD patients with PAH	down	hsa-miR-29b-2-5p/KCNB1	Modulates hypoxia-induced apoptosis, proliferation, and migration of human pulmonary artery smooth muscle cells (hPASMCs).	(Jin *et al.*, 2021) [21]
	circHACE1	Serum from smokers without or with COPD	up	miR-485-3p/TLR4	Aggregate CSE-triggered apoptosis, inflammation and oxidative stress in 16HBE cells.	(Zhou *et al.*, 2021) [22]
lncRNAs	ANRIL	Plasma from acute exacerbations of COPD patients	down	TNF-α, IL-1β, IL-17A, LTB-4	Associates with lower acute exacerbation risk, decreased inflammatory cytokines, and mild Chronic Obstructive Lung Disease (GOLD) stage.	(Ge *et al.*, 2019) [23]
	HOXA-AS2	Lung tissues from COPD patients	down	Notch1	Downregulation of HOXA-AS2 suppressed CSE-induced HPMECs proliferation.	(Zhou *et al.*, 2020) [24]
	LUCAT1	Human serum from COPD patients	up	miR-181a-5p/ Wnt/β- catenin	Promoted CSE-induced 16HBE cells apoptosis and inhibited proliferation.	(Zhao *et al.*, 2021) [25]
	MIAT	Mice model	up	miR-29c-3p/ HIF3A	Promoted CSE-induced murine lung epithelial cells and fibroblasts apoptosis, inflammation, and EMT.	(Gu *et al.*, 2022) [26]
	MEG3	Human peripheral blood from COPD patients	up	miR-149-3p/ NF-κB	Promoted CSE-induced 16HBE cells apoptosis.	(Lei *et al.*, 2021) [27]
	RP11-86H7.1	Cell model	up	miR-9-5p/ NF-κB	Promoted TRAPM2.5-induced 16HBE cells inflammation.	(Zhao *et al.*, 2020) [28]
	NNT-AS1	Human lung tissues from COPD patients	up	miR-582-5p/ FBXO11	Promoted CSE-induced 16HBE cells apoptosis, inflammation, and airway remodeling.	(Mei *et al.*, 2020) [29]
	XIST	Human lung tissues from COPD patients	up	miR-200c-3p/ EGR3	Promoted CSE-induced 16HBE cells apoptosis and inflammation.	(Chen *et al.*, 2021) [30]
	TUG1	Cell model	up	miR-9a-5p/ BCL2L11	Promoted CSE-induced HPMECs apoptosis.	(Chen *et al.*, 2021) [31]
	TUG1	Human induced-sputum and lung tissues from COPD patients	up	miR-145-5p/ DUSP6	Promoted CSE-induced 16HBE cells and lung fibroblasts inflammation and airway remodeling.	(Gu *et al.*, 2019) [32]
	TUG1	Lung tissues from COPD patients	up	α-SMA and fibronectins	TUG1 knockdown induced cellular viability following TGF-β stimulation in HFL1 cells.	(Tang *et al.*, 2016) [33]
	SNHG5	Human lung tissues from COPD patients	down	miR-132/PTEN	Inhibited CSE-induced 16HBE cells apoptosis and inflammation.	(Shen *et al.*, 2020) [34]
	CASC2	Human serum from COPD patients	down	miR-18a-5p/IGF1	Inhibited 16HBE cells apoptosis and inflammation.	(Liu *et al.*, 2021) [35]
	LINC00612	Human lung tissues from COPD patients	down	miR-31-5p/ Notch1	Inhibited CSE-induced HPMECs apoptosis, inflammation, and oxidative stress.	(Luo *et al.*, 2020) [36]
	NEAT1	Plasma from COPD patients	up	miR-193a	Positively correlates with GOLD stage, inflammation and predicts elevated COPD susceptibility and acute exacerbation risk.	(Ming *et al.*, 2019) [37]

Continued

Supplemental Table 1 Continued

Types	NcRNAs	Source	Expression	Target/pathway	Function	Study
lncRNAs	LOC729178	Human lung tissues from COPD patients	down	miR-144-3p/PHLPP2	Inhibited CSE-induced 16HBE cells inflammation.	(Wang *et al.*, 2021) [38]
	MIR155HG	Human lung tissues from COPD patients	up	miR-128-5p/BRD4	Promoted CSE-induced HPMECs apoptosis and inflammation.	(Song *et al.*, 2020) [39]
	LASI	Cynomolgus macaque model, and lung tissues from former smokers with and without COPD	up	None	LASI knockdown suppressed inflammation and mucus expression in CSE-treated HBECs.	(Manevski *et al.*, 2022) [40]
	CCAT1	Lung tissues of smokers with COPD	up	miR-152-3p/ERK	Promoted inflammation.	(Zong *et al.*, 2022) [41]

REFERENCES

1. Shen W, Liu J, Fan M, Wang S, Zhang Y, Wen L, *et al.* MiR-3202 protects smokers from chronic obstructive pulmonary disease through inhibiting FAIM2: An in vivo and in vitro study. Exp Cell Res 2018;362:370–77.
2. Sun Y, An N, Li J, Xia J, Tian Y, Zhao P, *et al.* miRNA-206 regulates human pulmonary microvascular endothelial cell apoptosis *via* targeting in chronic obstructive pulmonary disease. J Cell Biochem 2019;120:6223–36.
3. Wang D, He S, Liu B, Liu C. MiR-27-3p regulates TLR2/4-dependent mouse alveolar macrophage activation by targetting PPARγ. Clin Sci (Lond) 2018;132:943–58.
4. Du Y, Ding Y, Chen X, Mei Z, Ding H, Wu Y, *et al.* MicroRNA-181c inhibits cigarette smoke-induced chronic obstructive pulmonary disease by regulating CCN1 expression. Respir Res 2017;18:155.
5. Gu W, Yuan Y, Yang H, Wu H, Wang L, Tang Z, *et al.* Role of miR-195 in cigarette smoke-induced chronic obstructive pulmonary disease. Int Immunopharmacol 2018;55:49–54.
6. Leuenberger C, Schuoler C, Bye H, Mignan C, Rechsteiner T, Hillinger S, *et al.* MicroRNA-223 controls the expression of histone deacetylase 2: a novel axis in COPD. J Mol Med (Berl) 2016;94:725–34.
7. Shen W, Liu J, Zhao G, Fan M, Song G, Zhang Y, *et al.* Repression of Toll-like receptor-4 by microRNA-149-3p is associated with smoking-related COPD. Int J Chron Obstruct Pulmon Dis 2017;12:705–15.
8. Dang X, Yang L, Guo J, Hu H, Li F, Liu Y, *et al.* miR-145-5p is associated with smoke-related chronic obstructive pulmonary disease *via* targeting KLF5. Chem Biol Interact 2019;300:82–90.
9. Tang K, Zhao J, Xie J, Wang J. Decreased miR-29b expression is associated with airway inflammation in chronic obstructive pulmonary disease. Am J Physiol Lung Cell Mol Physiol 2019;316:L621–9.
10. Shen Z, Tang W, Guo J, Sun S. miR-483-5p plays a protective role in chronic obstructive pulmonary disease. Int J Mol Med 2017;40:193–200.
11. Zhong S, Chen C, Liu N, Yang L, Hu Z, Duan P, *et al.* Overexpression Of hsa-miR-664a-3p Is Associated With Cigarette Smoke-Induced Chronic Obstructive Pulmonary Disease *Via* Targeting FHL1. Int J Chron Obstruct Pulmon Dis 2019;14:2319–29.
12. Wu H, Ma H, Wang L, Zhang H, Lu L, Xiao T, *et al.* Regulation of lung epithelial cell senescence in smoking-induced COPD/emphysema by microR-125a-5p *via* Sp1 mediation of SIRT1/HIF-1a. Int J Biol Sci 2022;18:661–74.
13. Zhu M, Ye L, Zhu G, Zeng Y, Yang C, Cai H,*et al.* αROS-Responsive miR-150-5p Downregulation Contributes to Cigarette Smoke-Induced COPD *via* Targeting IRE1. Oxid Med Cell Longev 2022;2022:5695005.
14. Chen S, Yao Y, Lu S, Chen J, Yang G, Tu L, *et al.* CircRNA0001859, a new diagnostic and prognostic biomarkers for COPD and AECOPD. BMC Pulm Med 2020;20:311.
15. Li M, Hua Q, Shao Y, Zeng H, Liu Y, Diao Q, *et al.* Circular RNA circBbs9 promotes PM-induced lung inflammation in mice *via* NLRP3 inflammasome activation. Environ Int 2020;143:105976.
16. Zhou L, Wu B, Yang J, Wang B, Pan J, Xu D, *et al.* Knockdown of circFOXO3 ameliorates cigarette smoke-induced lung injury in mice. Respir Res 2021;22:294.
17. Zheng C, Zhang Y, Zhao Y, Duan Y, Mu Q, Wang X. Circ-OSBPL2 Contributes to Smoke-Related Chronic Obstructive Pulmonary Disease by Targeting miR-193a-5p/BRD4 Axis. Int J Chron Obstruct Pulmon Dis 2021;16:919–31.
18. Sun Y, Wu W, Zhao Q, Jiang R, Li J, Wang L, *et al.* CircGSAP regulates the cell cycle of pulmonary microvascular endothelial cells *via* the miR-942-5p sponge in pulmonary hypertension. Front Cell Dev Biol 2022;10:967708.
19. Miao Y, Wu J, Wu R, Wang E, Wang J. Circ_0040929 Serves as Promising Biomarker and Potential Target for Chronic Obstructive Pulmonary Disease. Int J Chron Obstruct Pulmon Dis, 2022;17:2079–92.
20. Ma H, Lu L, Xia H, Xiang Q, Sun J, Xue J, *et al.* Circ0061052 regulation

of FoxC1/Snail pathway *via* miR-515-5p is involved in the epithelial-mesenchymal transition of epithelial cells during cigarette smoke-induced airway remodeling. Sci Total Environ 2020;746:141181.
21. Jin X, Xu Y, Guo M, Sun Y, Ding J, Li L, *et al.* hsa_circNFXL1_009 modulates apoptosis, proliferation, migration, and potassium channel activation in pulmonary hypertension. Mol Ther Nucleic Acids 2021;23:1007–19.
22. Zhou F, Cao C, Chai H, Hong J, Zhu M. Circ-HACE1 Aggravates Cigarette Smoke Extract-Induced Injury in Human Bronchial Epithelial Cells *via* Regulating Toll-Like Receptor 4 by Sponging miR-485-3p. Int J Chron Obstruct Pulmon Dis 2021;16:1535–47.
23. Ge JL, Geng SS, Jiang H. Long noncoding RNAs antisense noncoding RNA in the INK4 locus (ANRIL) correlates with lower acute exacerbation risk, decreased inflammatory cytokines, and mild GOLD stage in patients with chronic obstructive pulmonary disease. J Clin Lab Anal 2019;33:e22678.
24. Zhou AY, Zhao YY, Zhou ZJ, Duan JX, Zhu YZ, Cai S, *et al.* Microarray Analysis of Long Non-Coding RNAs in Lung Tissues of Patients with COPD and HOXA-AS2 Promotes HPMECs Proliferation *via* Notch1. Int J Chron Obstruct Pulmon Dis 2020;15:2449–60.
25. Zhao S, Lin C, Yang T, Qian X, Lu J, Cheng J. Expression of long non-coding RNA LUCAT1 in patients with chronic obstructive pulmonary disease and its potential functions in regulating cigarette smoke extract-induced 16HBE cell proliferation and apoptosis. J Clin Lab Anal 2021;35:e23823.
26. Gu W, Wang L, Deng G, Gu X, Tang Z, Li S, *et al.* Knockdown of long noncoding RNA MIAT attenuates cigarette smoke-induced airway remodeling by downregulating miR-29c-3p-HIF3A axis. Toxicol Lett 2022;357:11–9.
27. Lei Z, Guo H, Zou S, Jiang J, Kui Y, Song J. Long non-coding RNA maternally expressed gene regulates cigarette smoke extract induced lung inflammation and human bronchial epithelial apoptosis *via* miR-149-3p. Exp Ther Med 2021;21:60.
28. Zhao J, Pu J, Hao B, Huang L, Chen J, Hong W, *et al.* LncRNA RP11-86H7.1 promotes airway inflammation induced by TRAPM2.5 by acting as a ceRNA of miRNA-9-5p to regulate NFKB1 in HBECS. Sci Rep 2020;10:11587.
29. Mei J, Zhang Y, Lu S, Wang J. Long non-coding RNA NNT-AS1 regulates proliferation, apoptosis, inflammation and airway remodeling of chronic obstructive pulmonary disease *via* targeting miR-582-5p/FBXO11 axis. Biomed Pharmacother, 2020, 129:110326.
30. Chen P, Jiang P, Chen J, Yang Y, Guo X. XIST promotes apoptosis and the inflammatory response in CSE-stimulated cells *via* the miR-200c-3p/EGR3 axis. BMC Pulm Med 2021;21:215.
31. Chen X, Mao M, Shen Y, Jiang X, Yin Z. lncRNA TUG1 regulates human pulmonary microvascular endothelial cell apoptosis *via* sponging of the miR-9a-5p/BCL2L11 axis in chronic obstructive pulmonary disease. Exp Ther Med 2021;22:906.
32. Gu W, Yuan Y, Wang L, Yang H, Li S, Tang Z, *et al.* Long non-coding RNA TUG1 promotes airway remodeling by suppressing the miR-145-5p/DUSP6 axis in cigarette smoke-induced COPD. J Cell Mol Med 2019;23:7200–9.
33. Tang W, Shen Z, Guo J, Sun S. Screening of long non-coding RNA and TUG1 inhibits proliferation with TGF-β induction in patients with COPD. Int J Chron Obstruct Pulmon Dis 2016;11:2951–64.
34. Shen Q, Zheng J, Wang X, Hu W, Jiang Y, Jiang Y. LncRNA SNHG5 regulates cell apoptosis and inflammation by miR-132/PTEN axis in COPD. Biomed Pharmacother 2020;126:110016.
35. Liu P, Zhang H, Zeng H, Meng Y, Gao H, Zhang M,*et al.* LncRNA CASC2 is involved in the development of chronic obstructive pulmonary disease *via* targeting miR-18a-5p/IGF1 axis. Ther Adv Respir Dis 2021;15:17534666211028072.
36. Luo J, Li L, Hu D, Zhang X. LINC00612/miR-31-5p/Notch1 Axis Regulates Apoptosis, Inflammation, and Oxidative Stress in Human Pulmonary Microvascular Endothelial Cells Induced by Cigarette Smoke Extract. Int J Chron Obstruct Pulmon Dis 2020;15:2049–60.
37. Ming XY, Duan WZ, Yi W. Long non-coding RNA NEAT1 predicts elevated chronic obstructive pulmonary disease (COPD) susceptibility and acute exacerbation risk, and correlates with higher disease severity, inflammation, and lower miR-193a in COPD patients. Int J Clin Exp Pathol 2019;12:2837–48.
38. Wang M, Liu Y, Zhang Y, Zhang L. LncRNA LOC729178 acts as a sponge of miR-144-3p to mitigate cigarette smoke extract-induced inflammatory injury *via* regulating PHLPP2 in 16HBE cells. J Mol Histol 2021;52:437–47.
39. Song J, Wang Q, Zong L. LncRNA MIR155HG contributes to smoke-related chronic obstructive pulmonary disease by targeting miR-128-5p/BRD4 axis. Biosci Rep 2020;40:BSR20192567.
40. Manevski M, Devadoss D, Long C, Singh SP, Nasser MW, Borchert GM, *et al.* Increased Expression of LASI lncRNA Regulates the Cigarette Smoke and COPD Associated Airway Inflammation and Mucous Cell Hyperplasia. Front Immunol 2022;13:988069.
41. Zong D, Liu X, Li J, Long Y, Ouyang R, Chen Y. LncRNA-CCAT1/miR-152-3p is involved in CSE-induced inflammation in HBE cells *via* regulating ERK signaling pathway. Int Immunopharmacol 2022;109:108818.
